# The Use of a Vancomycin-Eluting Calcium Sulfate and Hydroxyapatite Composite for Dead Space Management in a Fracture-Related Infection (FRI): A Retrospective Case Series

**DOI:** 10.7759/cureus.60390

**Published:** 2024-05-15

**Authors:** Olivia Mair, Magdalena Bonleitner, Philipp Rittstieg, Peter Biberthaler, Marc Hanschen

**Affiliations:** 1 Trauma Surgery, Klinikum rechts der Isar, Technical University Munich, Munich, DEU

**Keywords:** case series, cerament v, bone void filling, dead space management, fracture related infection

## Abstract

Background

Cerament V (CV) is a bioactive bone graft substitute with vancomycin as an antimicrobial agent designed for the management of bone defects and infections. In this retrospective case series, we aim to evaluate the clinical outcomes of patients treated with CV for fracture-related infections (FRI).

Methods

All patients who received treatment for FRI and whose dead space and bone reconstruction management was solely done utilizing CV were included. The patients were recruited between September 2015 and September 2022. Data including patient demographics, primary diagnosis, surgical procedure, antibiotic therapy, microbiological results, complications, and follow-ups were recorded. Outcomes were assessed, including the percentage of bone void filling on radiographs, infection resolution, adverse effects, and patient-reported outcome measures by EQ-5D-5L.

Results

We present in this retrospective case series seven patients (three female) with a mean age of 56.86 ± 16.27 years. All patients underwent surgical debridement and bone grafting using CV. Antibiotic therapy was tailored to the specific pathogens isolated in each case. Infection eradication was achieved in five patients. On average, new bone formation was 81% at six months and 99% at 12 months. Patient-reported outcome parameters (PROMs) utilizing the EQ-5D-5L questionnaire were recorded at a mean follow-up of 42.00 ± 27.97 months with a median EQ-5D-5L index of 0.541 (range: 0.459 - 0.97) and a mean EQ-5D-Visual Analogue Scale (VAS) score of 62.20 ± 24.68. No major adverse events related to CV were reported.

Conclusion

This retrospective case series demonstrates the potential efficacy of CV in managing FRIs. The bioactive and antibiotic properties of CV appear to facilitate infection resolution and bone healing, with an advantageous safety profile. Larger prospective studies are needed to further investigate the utility of CV in orthopedic practice.

## Introduction

Fracture-related infection (FRI) is a common and very challenging condition for surgeons worldwide. Since an international study group offered a clear definition of FRI standardizing diagnostic modalities as well as offering a treatment guideline in 2018, patient care and research quality have increased significantly [1-3].

Data from the literature suggests following defined surgical steps in the treatment of FRI, including tissue sampling, thorough debridement and excision of necrotic bone, dead space management, and reconstruction management via fracture stabilization and adequate soft tissue coverage [3,4]. Additionally, it has been shown that the application of local antimicrobial drugs should be strongly considered [5]. Resorbable and non-resorbable carriers of antimicrobials were suggested for dead space management. Polymethylmethacrylate (PMMA) has been used as a carrier for years and is well understood, but newer biodegradable carriers such as ceramics or bioglass have increasingly moved into focus [6-8]. In general, the advantage of the use of a resorbable antimicrobial material is that the treatment of FRI can be performed in a one-stage procedure; revision surgery with removal of the non-resorbable material and subsequent bone void filling with usually autologous bone grafts is not necessary anymore. A prerequisite for this single-stage treatment is that the antimicrobial material is highly osteoconductive and leads to the predictable generation of new bone to fill the bony defect [5].

In clinical practice, gentamycin, tobramycin, vancomycin, and clindamycin are most commonly used as local antibiotics. These drugs combine the specific need for a broad antimicrobial spectrum, especially in the gram-positive spectrum, a comparatively low resistance rate, good toxicity, and hypersensitivity profiles, and they work well with carriers [5].

One of these biodegradable ceramics is Cerament (CERAMENT BONE VOID FILLER, Bonesupport, Lund, Sweden), a combination of calcium sulfate (CaS) and hydroxyapatite (HA), which has shown high osteoconductivity in bone voids after tibial plateau fractures [9]. The addition of gentamicin (CG, CERAMENT G, Bonesupport, Lund, Sweden) can protect bone healing from gentamicin-sensitive microorganisms and has shown excellent results in FRI and osteomyelitis [4,10,11]. While CG has been investigated well in recent years, data on CG’s sister product, CV (CV, CERAMENT V, Bonesupport, Lund, Sweden), where vancomycin is added to CaS/HA, is rather scarce.

Gentamycin is an aminoglycoside antibiotic. It is bactericidal by disrupting the protein and is active mostly against gram-negative bacteria, including *Pseudomonas, Proteus, Escherichia coli, Klebsiella pneumoniae, Enterobacter aerogenes, Serratia*, and the gram-positive *Staphylococcus* [12].

Vancomycin is a glycopeptide antibiotic. It is bactericidal by inhibiting the bacterial cell wall synthesis of gram-positive bacteria such as *Staphylococcus, Streptococcus, Enterococcus, Pneumococcus, *and* Clostridia*. FRI and osteomyelitis are mainly caused by gram-positive bacteria, making vancomycin a highly viable option for the management of FRI [12].

As clinical research on CV is still scarce, this case series aims to evaluate the efficacy and outcome after using CV in FRI and osteomyelitis. Additionally, this case series aims to evaluate whether the clinical and radiographical outcome of CV in FRI is comparable to the results of CG in the literature.

## Materials and methods

The study was conducted at the Klinikum rechts der Isar, Technical University Munich, Germany. We conducted a retrospective, single-center evaluation of all patients who underwent surgery for FRI and who were solely treated with CV for dead space management and bone reconstruction management between September 2015 and September 2022 in a level 1 trauma center. Local ethics committee approval was obtained (application number 2022-57-S-NP).

Patients were excluded when a combination of different local antibiotics was used, when the minimum clinical follow-up was under 12 months, or when the minimal radiographic follow-up was under six months. Additionally, we excluded patients under 18 years of age and those who were pregnant.

Standardized treatment protocol

Systemic antibiotic therapy was stopped in all patients at least two weeks prior to surgery, provided it was safe to do so. All patients were treated according to a single-stage protocol for FRI [10,12]. After an open approach with a good overview of the surgical site, multiple deep samples were taken using an established protocol. After samples had been taken, intravenous antibiotics were given. Sinus tracts were excised and contaminated implants removed, followed by the excision of necrotic bone, continuing until healthy, bleeding bone was exposed. The area was then irrigated, and the cavity was dried by packing it with gauze. If instability was present, stabilization was provided by external fixation. Postoperatively, patients were treated with intravenous antibiotics according to pre-operative antimicrobial susceptibility testing (AST) when possible, or with vancomycin (initially 1g every 12 hours) and meropenem (500mg every eight hours) until the results of the new samples were available.

CV was used for dead space and bone reconstruction management. No additional material or antibiotic was added, and primary skin closure was achieved directly or by local or free microvascular muscle flaps.

Data collection

Demographics, comorbidities, etiology of infection, clinical symptoms, diagnostic tests, antibiotic treatment choice, and duration were collected from the patient charts. Additionally, soft tissue contamination, soft tissue status, and vascularity were noted. The extent of bony involvement (Cierny-Mader (CM) anatomical type) was determined according to the surgical notes. Radiological and microbiological outcomes were confirmed by two assessors who were not involved in the management of the patients.

Clinical outcome parameters

The primary outcome parameter was infection eradication at a minimum of one year after surgery. It was defined as the absence of further surgery performed for infection, no requirement for new systemic antibiotic treatment due to the new onset of symptoms, the absence of recurrent infection with positive cultures from further aspiration or biopsy, and no recurrent sinus formation [10,12].

Secondary outcome parameters were delayed wound healing or persistent wound drainage, any procedure or intervention required to manage wound problems after initial surgery, any systemic reaction to CV, pathological fracture at the site of surgery, and the occurrence of mortality.

Radiographic outcome parameters

**Figure 1:**
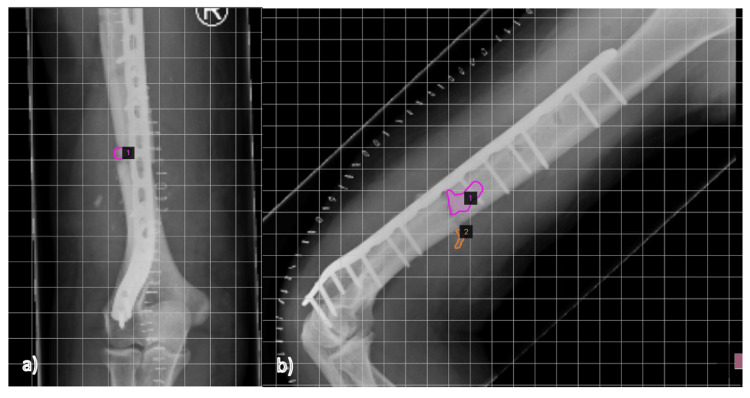
Exemplary measurement of bone void filling with CV on the SketchAndCalc Area Calculator

Radiographic evaluation was performed using the method described by Ferguson et al. [13]. In short, orthogonal postoperative images were examined to delineate the extent of the bone void filled with CV and compared to their follow-up radiographs. SketchAndCalc Area Calculator software was used to determine the percentage of new bone formation at six- and 12-month follow-ups (Figure 1).

Once radiographs were imported into the software, images were calibrated using the area calculator's auto-scale tool. The software’s area calculator app and image magnification were used to freehand-draw the perimeter of the space filled by CV immediately after surgery (i.e., the initial bone graft substitue (BGS) area) and the unfilled or residual bone void at the final follow-up. The following formula was used to calculate the new bone formation on each view: [(initial CV surface area - residual bone-void) / initial CV surface area].

This process was done for both anteroposterior and lateral views. These two percentage scores were added together and divided by two to give the final mean percentage of new bone formation. The percentage of new bone formation in the space that was initially filled by CV was measured to the nearest 5%.

Patient-reported outcome parameters (PROMs)

PROMs were collected using the EQ-5D-5L questionnaire. The EQ-5D-5L is a self-assessed, health-related quality of life questionnaire. The scale measures the quality of life in five categories, including mobility, self-care, usual activities, pain or discomfort, and anxiety or depression, each of which has five levels of responses. The EQ-5D-5L index can then be calculated based on the validated conversion model specific to Germany. An index of 1 represents a perfect health state, whereas an index of -0.594 represents the worst health condition [14].

Part two of the survey is a Visual Analogue Scale (VAS), in which respondents rate their perceived health from 0 (worst possible health) to 100 (best possible health) [15]. It has been evaluated and validated in a German population and is available in the German language [14].

## Results

We were able to identify seven patients who fit our strict inclusion criteria. There were three (42.9%) female patients, and the average age at the time of surgery was 56.86 ± 16.27 years.

The Cierny-Mader classification defined three patients as CM type III and four patients as CM type IV [16]. An overview of the patient demographics, primary diagnosis, anatomical location, etiology of infection, and CM type can be found in Table 1.

**Table 1:** Patient demographics and specification of diagnosis ASA: American Society of Anesthesiologists; CV: Cerament V; F: female; M: male; ORIF: open reduction and internal fixation; HAN: hindfoot arthrodesis nail

Patient - ID	Age (years)	Sex	ASA	Anatomical location	Cierny- Mader classification	Primary diagnosis	Type of surgery when CV was used
1	84	F	IV	Tibial shaft	3	Non-union and deep tissue infection after ORIF of tibial shaft fracture (e.d.)	Intramedullary reaming and application of CV
2	64	F	II	Proximal femur	4	Non-union fracture after intramedullary nailing of proximal femoral fracture (e.d.)	Removal of intramedullary nail, radical debridement, bone void filling with CV, plate osteosynthesis
3	66	M	II	Calcaneus	3	Deep wound infection after ORIF of the calcaneus	Removal of the plate, filling of screw holes with CV
4	42	M	II	Distal tibia	4	Implant failure of HAN-nail after multiple revision surgeries after °III open tibial pilon fracture	Removal of HAN-nail, intramedullary application of CV
5	49	F	I	Proximal femur	4	Non-union after intramedullary nailing of proximal femoral fracture	Intramedullary nailing with a coating of the nail with CV and bone void filling
6	36	M	I	Distal fibula	3	Deep wound infection after removal of a set screw	Removal of plate, filling of screw holes with CV
7	57	M	II	Humeral shaft	4	°III open fracture	ORIF humeral shaft + application of CV in fracture

Pre-operative microbiology results and antimicrobial susceptibility testing (AST) from the previous treatments were available in four cases. *Propionibacterium* *acnes* was identified in one patient, *Staphylococcus hominis* and *Propionibacterium acnes* in one, *Staphylococcus epidermidis* in one, and *Pantoea agglomerans* in one pre-operatively. 

Intraoperatively, *Staphylococcus aureus* was found in two patients, *Staphylococcus hominis* in one, *Propionibacterium acnes* in one, and *Staphylococcus epidermidis* and *Propionibacterium acnes* in one. Two patients had negative intraoperative microbiological results. Table 2 shows pre- and intraoperative microbiology, gram-stain, and calculated and targeted antibiotic therapy.

**Table 2:** Pre- and intraoperative microbiology, gram-stain, and primary and targeted antibiotic therapy AST: antimicrobial susceptibility testing; +: gram positive; -: gram-negative

Patient- ID	Pre-operative microbiology	Pre-operative AST available?	Gram-stain	Intra-operative microbiology	Calculated antibiotic therapy	Targeted antibiotic therapy
1	Propionibacterium acnes	yes	+	Staphylococcus hominis	Meropenem + Vancomycin	Meropenem
2	n.a.	no	n.a.	Propionibacterium acnes	none	Clindamycin
3	n.a.	no	n.a.	Staphylococcus aureus	Meropenem + Vancomycin	Flucloxacillin
4	Staphylococcus epidermidis	yes	+	none	Ampicillin/ Sulbactam	none
5	Staphylococcus hominis + Propionibacterium acnes	yes	+ +	Staphylococcus epidermidis + Propionibacterium acnes	none	Clindamycin + Cefuroxime
6	n.a.	no	n.a.	Staphylococcus aureus	Ampicillin/ Sulbactam	Cefazolin
7	Pantoea agglomerans	yes	-	none	Cefuroxime	None

Outcome

Eradication of infection was achieved in six (85.7%) of the seven patients. One patient (14.3%) underwent amputation of the leg after 30 months due to a recurrence of infection. We did not record any pathologic fractures or systemic reactions to CV. Two patients (28.6%) had persistent wound discharge up to three months after surgery, with one patient (14.3%) requiring revision surgery after three months. 

The mean percentage of new bone formation was 81% (n= 5) at six months and 99% (n= 6) at 12 months. The individual measurements can be found in Table 3.

**Table 3:** Radiographic outcome Percentage of new bone formation and clinical outcome with PROMs CV: Cerament V; AST: antimicrobial susceptibility testing; VAS: Visual Analogue Scale; PROMs: patient-reported outcome parameters

Patient - ID	Radiological outcome: percentage of bone void filling		Complications (if yes, specification)	Primary outcome parameters			EQ-5D-5L - Response	EQ-5D-5L index value	EQ- VAS score	Time of clinical follow-up (months)
	At six-month follow-up (in %)	At 12-month follow-up (in %)		Revision surgery	Eradication of infection after 12- months	Adverse effects				
1	n.a.	95	no	no	yes	No	n.a.	n.a.	n.a.	n.a.
2	80	100	No	no	yes	No	11112	0.97	80	19
3	90	n.a.	Yes, persistent wound discharge up to three months postoperatively	Yes, three months after the CV-surgery	yes	No	41334	0.459	30	16
4	65	100	Yes, wound discharge six weeks postop.	no	Amputation (30 months after CV)	No	43433	0.485	45	65
5	90	100	no	no	yes	No	41433	0.541	66	78
6	n.a.	100	no	no	yes	No	n.a.	n.a.	n.a.	n.a.
7	80	100	no	no	yes	No	11211	0.964	90	32

We were able to contact five patients to evaluate patient satisfaction. One patient (14.3%) was deceased 17 months after surgery due to unconnected reasons, and we were not able to reach one other patient (14.3%). 

The median EQ-5D-5L index was 0.541 (range: 0.459 - 0.97), and the mean EQ-5D-VAS score was 62.20 ± 24.68. The mean follow-up time at collection of PROMs was 42.00 ± 27.97 months.

Exemplary case presentations

Patient 2

A 64-year-old female patient presented with progressive immobilizing pain during the last four weeks without trauma. The X-ray showed a fracture of the long femoral neck on the left side at the level of the locking screws with consecutively displaced nails, as well as the neck and trochanter major. Due to the atraumatic fracture of the femoral neck and the implant, a FRI was suspected. The following surgery was performed: removal of the existing femoral nail, radical debridement and medullary canal reaming, plate osteosynthesis, autologous cancellous bone grafting from the ipsilateral iliac crest, allogeneic cancellous bone grafting, CV implantation, and re-osteosynthesis with a long cephalomedullary nail. The microbiological results showed *Propionibacterium* *acnes* in two of eight tests. Therefore, the antibiotic therapy was postoperatively continued with clindamycin according to the AST for 10 days. A follow-up radiograph at 12 months shows complete bone healing and almost complete remodeling of the CV (96% ap and 100% lat; summary score 98%). PROMs can be seen in Table 3.

Patient 7

A 57-year-old male patient presented with FRI after a humeral shaft fracture and external fixation three weeks prior in another hospital. Pre-operative microbiology from the previous hospital showed *Pantoea agglomerans* in two of five samples. He had been treated with oral ciprofloxacin for the past three weeks. Therefore, a two-staged approach was planned. Two weeks prior to surgery, the oral antibiotics were stopped. The patient thereafter did not show any signs of infection. In the first surgery, thorough irrigation was done, and multiple samples from soft tissue and bone were taken. The microbiological results were negative, which is why in a second surgery ORIF was done (10-hole LCP plate, Synthes) and CV was used for dead space and bone reconstruction management. The skin was closed primarily (Figure 1). A follow-up radiograph at three months showed fracture consolidation with ongoing remodeling of CV into bone. The final follow-up radiograph at 12 months shows complete bone healing and almost complete remodeling of the CV (96% ap and 100% lat; summary score 98%). PROMs can be seen in Table 3.

## Discussion

To the best of our knowledge, this is the first case series on FRI in which only CV was used for dead space management and treatment of local infections.

Since the development of clear guidelines by an international study group in 2018, the treatment of FRI has improved significantly. The study group also suggests the use of local antimicrobial drugs, preferably linked to resorbable and non-resorbable carriers [5]. The clear advantage of using local antibiotic carriers is their ability to elute high local doses of antibiotics without having a systemic effect on the patient [13].

As Cerament is a highly osteoconductive material that has routinely been used for dead space management with very good success, the addition of local antibiotics is very promising. So far, we have mainly used CG for dead space and bone defect management of FRI, with the rationale that gentamicin is active against gram-negative and gram-positive bacteria. Sometimes a pre-op sampling has not been performed or is not reliable, so we assumed gentamicin was the safer choice. However, most FRI are caused by gram-positive bacteria, *Staphylococcus aureus* is the most frequent microorganism in any type of osteomyelitis, and vancomycin is very often used as a local antibiotic in the treatment and prophylaxis of bone infections [1,17]. Therefore, we started to use CV in selected cases, usually where a pre-op sample was available or the patient’s history suggested a high likelihood of a gram-positive infection. 

With this case series, we aimed to evaluate the safety, efficacy, and outcome after the use of CV in FRI as well as its value when compared to similar materials. As no adverse reactions were noted in our patients, safety seems to be of no concern in the use of CV. In our small series, the mean new bone formation was 81% at six months and 99% at 12 months. Using the same method of measurement, Ferguson et al. reported a percentage of new bone formation of 73.9% in their retrospective review of a prospectively collected database of 180 subjects treated with CG for chronic osteomyelitis [18]. Therefore, the outcome of bone-void healing from the standalone use of CV seems comparable to the results of CG.

Six patients showed eradication of infection at 12-month follow-up, with one patient requiring revision surgery with irrigating and wound revision for persistent wound discharge three months after the initial surgery. One patient (14.3%) never reached eradication of infection and required amputation (in Burgess’ technique) 30 months after treatment with CV. In larger case series on the use of CG for similar surgical indications, a recurrence rate of 7-9% has been reported [4,10,13,19]. Admittedly, our case series on CV is smaller and we are reporting early experiences; nevertheless, so far, the outcome of the stand-alone use of CV is comparable with the results of CG. 

The patient's reported outcome was significantly lower on the EQ-5D-5L score and EQ-VAS core than that of the general German population. These results are similar to those presented by other studies concerning the treatment of patients with osteomyelitis and FRI [20,21]. However, we do not think that the low outcome in terms of PROMs is related to the use of CV but rather to the severe impact chronic diseases such as FRI and osteomyelitis have on patients’ lives.

This case series has several limitations. First of all, we present a small sample size, which will not allow for general assumptions after the sole use of CV in FRI. As uniform guidelines were only presented in 2018 by an international study group, patients who were treated before 2018 did not undergo a standardized treatment protocol in our hospital. 

Due to the retrospective nature of this case series, information about pre-operative health status and PROMs is not available, and the possibility of missing or incorrect data in patient reports is always present. Also, there could be bias in this study due to missing or incomplete patient data. 

As knowledge is still limited, we believe that further, larger prospective trials will be warranted, as CV seems to have great value in terms of safety, efficacy, and outcome on FRI.

## Conclusions

From our early experience, it seems that the standalone use of CV for dead space and bone reconstruction management in FRI has a comparable outcome to the use of CG concerning the recurrence of infection and new bone formation in selected cases, especially when pre-operative microbiology is available.
